# Novel compounds protect auditory hair cells against gentamycin-induced apoptosis by maintaining the expression level of H3K4me2

**DOI:** 10.1080/10717544.2018.1461277

**Published:** 2018-04-24

**Authors:** Ao Li, Dan You, Wenyan Li, Yingjie Cui, Yingzi He, Wen Li, Yan Chen, Xiao Feng, Shan Sun, Renjie Chai, Huawei Li

**Affiliations:** a ENT Institute and Otorhinolaryngology Department of Affiliated Eye and ENT Hospital, Key Laboratory of Hearing Medicine of NHFPC, Shanghai Engineering Research Centre of Cochlear Implant, State Key Laboratory of Medical Neurobiology, Fudan University, Shanghai, China;; b Department of Otorhinolaryngology Head and Neck Surgery, Affiliated Drum Tower Hospital of Nanjing University Medical School, Research Institution of Otorhinolaryngology, Jiangsu Provincial Key Medical Discipline (Laboratory), Nanjing, China;; c Knowshine (Shanghai) Pharmaceuticals Inc, Shanghai, China;; d Key Laboratory for Developmental Genes and Human Disease, Ministry of Education, Jiangsu Province High-Tech Key Laboratory for Bio-Medical Research, Institute of Life Sciences, Southeast University, Nanjing, China;; e Co-innovation Center of Neuroregeneration, Nantong University, Nantong, China;; f Institutes of Biomedical Sciences and The Institutes of Brain Science and the Collaborative Innovation Center for Brain Science, Fudan University, Shanghai, China

**Keywords:** Apoptosis, gentamycin, H3K4me2, hair cell, compounds

## Abstract

Aminoglycoside-induced hair cell (HC) loss is a major cause of hearing impairment, and the effective prevention of HC loss remains an unmet medical need. Epigenetic mechanisms have been reported to be involved in protecting cochlear cells against ototoxic drug injury, and in this study we developed new bioactive compounds that have similar chemical structures as the epigenetics-related lysine-specific demethylase 1 (LSD1) inhibitors. LSD1 inhibitors have been reported to protect cochlear cells by preventing demethylation of dimethylated histone H3K4 (H3K4me2). To determine whether these new compounds exert similar protective effects on HCs, we treated mouse cochlear explant cultures with the new compounds together with gentamycin. There was a severe loss of HCs in the organ of Corti after gentamycin exposure, while co-treatment with the new compounds significantly protected against gentamycin-induced HC loss. H3K4me2 levels in the nuclei of HCs decreased after exposure to gentamycin, but H3K4me2 levels were maintained in the presence of the new compounds. Apoptosis is also involved in the injury process, and the new compounds protected the inner ear HCs against apoptosis by reducing caspase-3 activation. Together, our findings demonstrate that our new compounds prevent gentamycin-induced HC loss by preventing the demethylation of H3K4me2 and by inhibiting apoptosis, and these results might provide the theoretical basis for novel drug development for hearing protection.

## Introduction

In all mammals, hair cells (HCs) are the sensory receptors of both the auditory and the vestibular systems (Borg & Viberg, 2002). Because HCs are terminally differentiated and are incapable of regeneration, damage to HCs results in permanently reduced hearing sensitivity and in severe cases to complete sensorineural hearing loss (Henley & Rybak, 1995). Sensorineural hearing loss is a common health problem worldwide, and a significant proportion of hearing loss is caused by the use of aminoglycoside antibiotics, with reported incidences of hearing loss in 2–25% of treated patients (Huth et al., 2011). Aminoglycosides such as gentamycin (also known as gentamicin), kanamycin, and neomycin are commonly prescribed antibiotics for the treatment of severe gram-negative bacterial infections (Schatz et al., 2005), but the clinical use of aminoglycosides is restricted by severe ototoxic side effects that can lead to the loss of HCs (Durante-Mangoni et al., 2009). In recent years, many new compounds have been developed to understand the ototoxicity and mechanisms of HC injury induced by aminoglycosides as a way to protect HCs from damage (Dong et al., 2015; Glutz et al., 2015).

Epigenetic modifications play an important role in the regulation of many chromosomal functions and are closely linked to certain biological events such as transcriptional regulation, cancer development, and cell death (Reik, 2007; Greer & Shi, 2012; Rizzi et al., 2012). More recently, epigenetic factors and non-coding RNAs have emerged as an additional layer of gene regulation in the hearing research field (Doetzlhofer & Avraham, 2017). In inner ear development, recent studies have shown that histone deacetylases 1 and 3 (HDAC1 and HDAC3) are expressed in the developing otic vesicles and play an important role in otic vesicle formation. Knockdown of HDAC1 or HDAC3 in zebrafish embryos induces smaller otic vesicles, abnormal otoliths, malformed or absent semicircular canals, and fewer sensory HCs (He et al., 2016a, 2016b), indicating that epigenetic regulation plays crucial roles in the development of auditory organs. Previous studies of inner ear disease and injury have shown that after exposure to traumatic noise histone H3 lysine 9 acetylation (H3K9ac) is decreased in the nuclei of outer HCs (OHCs), while HDAC1, HDAC2, and HDAC3 are increased in OHCs, and that HDAC inhibitors can significantly reduce OHC loss and attenuate noise-induced hearing loss (Chen et al., 2016). Histone methylation is a major covalent modification that epigenetically regulates cell-specific gene expression patterns (Shi et al., 2004; Wang et al., 2011). Recent studies have highlighted specific functional roles of histone methylation in a variety of cellular processes such as cell differentiation, survival, and death (Rugg-Gunn et al., 2010; Greer & Shi, 2012). Our previous studies showed that the G9a inhibitor BIX01294 protects against neomycin-induced HC loss by inhibiting dimethylation of H3K9 (Yu et al., 2013).

Dimethylation of histone H3K4 (H3K4me2) is an important histone marker that is frequently associated with gene activation, and the distribution of H3K4me2 is dynamic throughout all stages of cell differentiation and tissue development (Popova et al., 2012; Zhang et al., 2012; Lei et al., 2015). The histone demethylase LSD1 was reported to repress transcription primarily by demethylating H3K4me2 (Shi et al., 2004), and studies on LSD1 have identified several inhibitors, such as tranylcypromine, S2101, and CBB1007, that efficiently block LSD1-mediated demethylation of H3K4me2 (Yang et al., 2007; Wang et al., 2009). Using zebrafish and mouse models to screen small-molecule inhibitors of LSD1, we have previously shown that some LSD1 inhibitors protect cochlear HCs and spiral ganglion neurons (SGNs) against ototoxicity-induced death by maintaining H3K4me2 levels (He et al., 2014b, 2015; Li et al., 2015a).

The development of therapeutic drugs that protect HCs against aminoglycoside-induced ototoxicity is an unmet medical need because these antibiotics are still necessary to combat severe infections. In this study, we developed a set of novel compounds that have similar chemical structures as LSD1 inhibitors and investigated their otoprotective effect against gentamycin-induced ototoxicity *in vitro*. S2101, a known LSD1 inhibitor, served as a positive control in our present studies. We show here that the level of H3K4me2 decreased following gentamycin-induced HC damage and that, similar to S2101, our new compounds maintained high levels of H3K4me2 and prevented HC apoptosis. Based on these findings, we suggest that these new compounds are novel agents for protecting HCs against gentamycin-induced ototoxicity.

## Materials and methods

### Cochlear organotypic explant cultures

Animal experiments were carried out in strict accordance with the ‘Guiding Directive for Humane Treatment of Laboratory Animals’ issued by the Chinese National Ministry of Science and Technology in September 2006. All experiments were approved by the Shanghai Medical Experimental Animal Administrative Committee (Permit Number: 2009-0082), and all efforts were made to minimize suffering and reduce the number of animals used. Cochleae of 2-day-old postnatal C57BL/6 mice were dissected in Hanks solution (Invitrogen, Carlsbad, CA). To obtain a flat basilar membrane surface, the spiral ganglion, Reissner’s membrane, and the stria vascularis were carefully removed. Cochlear explants were plated onto 35 mm^2^ dishes coated with poly-l-lysine (Sigma-Aldrich, St. Louis, MO) and cultured in DMEM/F12 medium with N2/B27 supplement (Invitrogen). After the cochlear explants were attached to the dishes, the culture medium was removed and replaced with medium containing the different compounds. In different culture experiments, 1 mM gentamycin (Sigma-Aldrich), 20 μM compound A, 20 μM compound B, or 20 μM of the LSD1 inhibitor S2101 (Calbiochem; Merck Millipore, Bedford, MA) were added as indicated in the text. The new compounds were dissolved in water and S2101 was dissolved in DMSO at stock concentrations of 20 mM and then diluted to their final concentrations in culture medium.

### Design and synthesis of the novel compounds

**Figure 1.**
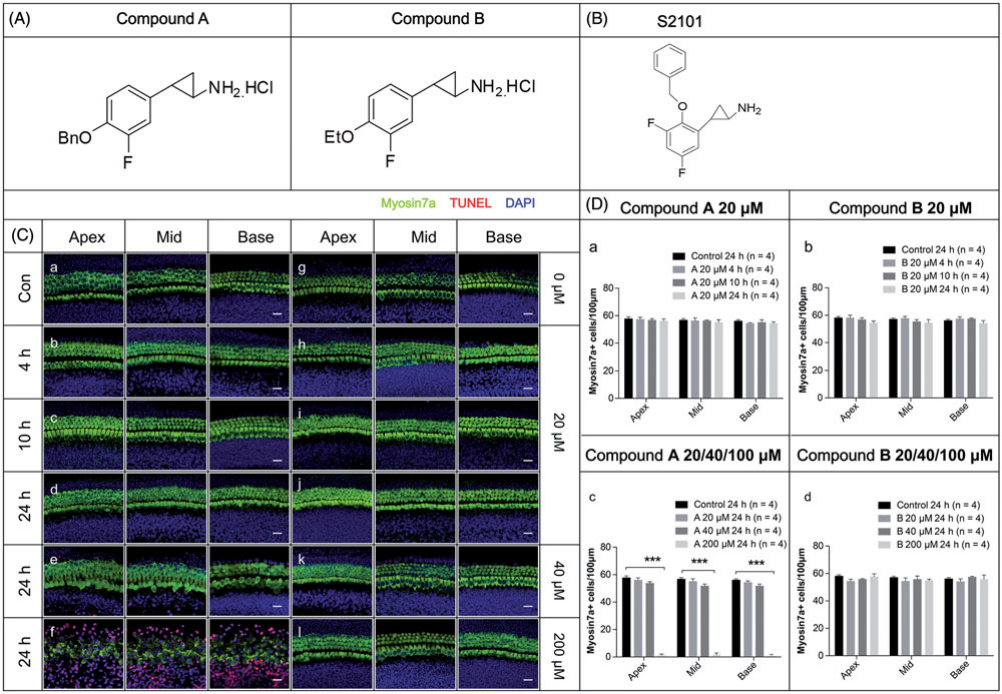
The structures and the cytotoxicity of the two new compounds. (A–B) The structures of the two new compounds (A) and S2101 (B). (C) The cytotoxicity of the two new compounds. Confocal images of the apical, middle, and basal turns in the cochlear explant cultures with only compound A (b–f) or B (h–l) for 4 h, 12 h, or 24 h. Normal tissues with no compound were used as controls (a, g). No cytotoxicity to the *in vitro* cochlear explants was observed at 20 μM compound A (b–d) or B (h–l) from 4 h to 24 h. At 40 μM compound A (e) and B (k) for 24 h, the cochlear explants showed no obvious change in morphology or the number of HCs. When treating the cochlear explants with 200 μM compound A for 24 h, the number of HCs decreased from the apical to basal turn, the HCs exhibited altered morphologies, and numerous TUNEL-positive cells were seen (f). There were no obvious changes in the morphology or arrangement of HCs when treating the cochlear explants with 200 μM compound B for 24 h (l). The HCs were labeled with myosin VIIa antibody (green), and the nuclei were stained with DAPI (blue). Apoptotic cells were labeled with TUNEL (red). (D) The quantification of HCs treated with different concentrations of the compounds at different time points. The HC numbers in the three different turns of the cochlear cultures treated with 20 μM compound A (a) or compound B (b) for 4 h, 10 h, or 24 h are shown in the bar charts. HC numbers in the cochlear cultures treated with 20 μM, 40 μM, and 200 μM compound A (c) and B (d) for 24 h are shown in the bar charts. Four cochleae were used for each group. Data are expressed as the mean ± S.E. ***p < .01 vs. the 20 μM compound A group by one-way ANOVA.

The LSD1 inhibitor S2101 has been shown to protect against drug-induced HC loss in our previous research (He et al., 2015). Here we used S2101 as the positive control to show the protective effects of LSD1 inhibitors on HCs after gentamycin exposure. To explore the mechanism behind the effects of this type of compound, we designed and synthesized two new derivatives of S2101. The fluorine atom was retained in both of the compounds, and as hydrochloride salts these compounds showed good solubility in water. The structures of the two new compounds are shown in Figure 1(A), and the structure of S2101 is shown in Figure 1(B).

**Figure 2.**
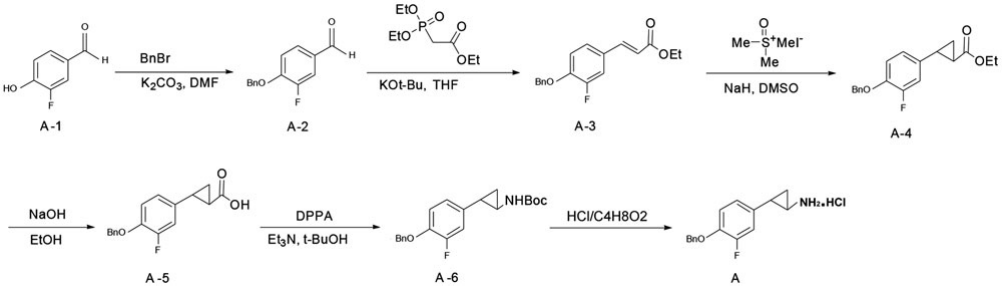
The synthesis of the new compounds (using compound A as an example).

The synthesis of the new compounds used conventional solution chemistry as shown in Scheme 1 using Compound A as an example (Figure 2). 3-Fluoro-4-hydroxybenzaldehyde (A-1) was converted into 4-(benzyloxy)-3-fluorobenzaldehyde (A-2), which was used in the next step without further purification. A condensation of intermediate A1-2 and Wittig reagent gave the alkene A-3 with a 93% yield. The alkene was treated with trimethylsulfonium iodide to give cyclopropanecarboxylate (A-4) with a 40% yield, and this was hydrolyzed to produce an acid (A-5) that was used in the next step without further purification. The acid was treated with diphenylphosphoryl azide (DPPA) in a Curtis rearrangement reaction to produce tert-butyl 2-(4-(benzyloxy)-3-fluorophenyl) cyclopropylcarbamate (A-6) with a 40.3% yield. The Boc protecting group in compound A1-6 was removed by the action of hydrochloride to furnish compound A with a 56% yield. The preparation of compound B was conducted according to the procedures identical to those of compound A except for a different substituted group (Figure 1(A)).

### Immunofluorescence

Organotypic cultures (including a control group) fixed in 4% paraformaldehyde were rinsed three times with phosphate buffered saline (PBS) and permeabilized with 0.2% Triton X-100 in PBS for 30 min at room temperature. Permeabilized samples were then blocked with 10% donkey serum in PBS for 1 h and incubated with primary antibody at 4 °C overnight. Nuclei were labeled with 4′,6-diamidino-2-phenylindole (DAPI) for 10 min at room temperature. The following primary antibodies were used: anti-H3K4me2 (1:500 dilution; Abcam, Cambridge, MA), anti-cleaved caspase-3 (1:200 dilution; Cell Signaling Technology, Inc., Danvers, MA), and anti-myosin VIIa (1:400 dilution; Proteus Biosciences, Ramona, CA). After unbound antibodies were washed away, the tissues were incubated with the corresponding secondary antibodies conjugated with tetramethylrhodamine (TRITC) or fluorescein isothiocyanate (FITC) (1:400 dilution; Jackson ImmunoResearch, West Grove, PA). The images were captured using a Leica SP5 confocal microscope with a Leica microsystems LAF AF-TCS MP5 2.6.0 system (Leica, Heidelberg, Germany).

### Western blot analysis

Total protein was isolated from a pool of 10 cultured cochleae in each group using the AllPrep DNA/RNA/Protein Mini Kit (QIAGEN, Hilden, Germany) according to the manufacturer’s instructions. A BCA protein kit (Beyotime, Jiangsu, China) was used to measure the protein concentrations. After separation by 12% SDS-PAGE, the proteins were blotted onto PVDF membranes (Immobilon-140 P; Millipore, Bedford, MA). The membranes were blocked with 5% nonfat dried milk in TBST (50 mM Tris-HCl pH 7.4, 150 mM NaCl, and 0.1% Tween-20) for 1 h at room temperature and hybridized overnight at 4 °C with the primary antibodies. Antibodies included anti-H3K4me2 (1:1000 dilution; Abcam), anti-cleaved caspase-3 (1:500 dilution; Cell Signaling Technology, Inc.), and anti-GAPDH (1:5000 dilution; Cell Signaling Technology, Inc.). The membranes were subsequently washed three times in Tris-buffered saline with Tween-20 (TBST) for 10 min each and then incubated with HRP-conjugated secondary antibody diluted 1:5000 (Supersignal West, Pierce, Rockford, IL) for 1 h at room temperature. Immunoreactive bands were visualized using the ECL-Kit according to the manufacturer’s instructions (Pierce).

### Quantitative analysis

To quantify the number of surviving HCs in the organ of Corti, the explant was divided into three segments of equal length from the apex to the base (apical, middle, and basal turns). The numbers of HCs that were positively stained with the anti-myosin VIIa antibody were counted.

### TUNEL staining

Apoptotic cells in the organ of Corti were detected by TUNEL assay according to the manufacturer’s protocol. Briefly, after washing with PBS, the samples were incubated with TUNEL reaction mixture (Roche, Switzerland) in a humidified chamber at 37 °C for 60 min and finally labeled with DAPI to visualize the nuclei.

### FM1-43FX labeling of cochlear HCs

To measure the effect of blocking mechanotransduction, FM1-43FX (Molecular Probes, Eugene, OR) uptake into cochlear HCs was examined. The cochlear organs from the control group and the treatment groups were incubated with serum-free medium containing FM1-43FX for 3 min. The cochleae were then rinsed twice in serum-free medium and fixed for analysis by fluorescent microscopy.

### Statistical analysis

Comparisons of the average values between two groups were analyzed using Student’s *t*-test or ANOVA with multiple comparisons using SigmaPlot version 12.5. All values are presented as the mean ± standard error. *p* Values less than .05 were considered statistically significant.

## Results

### Safety and toxicity of compounds a and B

Explants of the organs of Corti from postnatal Day 2 mice were used to determine the toxicity of the new compounds. The cochlear explants were cultured with either compound A or compound B at different concentrations from 20 µM to 200 μM for 4 h to 24 h. With the regular working concentrations, such as 20 µM or 40 μM, we found that after 4 h, 10 h, and even 24 h culture, the explants maintained good structures with all of the HCs showing normal morphologies, and no TUNEL-positive cells were observed (Figure 1C (b–e), (h–k), D). At a very high concentration of 200 μM for 24 h, large numbers of TUNEL-positive cells were detected in the compound A group along with significant HC loss and disorganization of the cochlear structure (Figure 1Cf, D). However, the explants cultured in 200 μM compound B for 24 h remained relatively intact, with no obvious HC loss or disorganized cochlear structure (Figure 1Cl, D). These results demonstrated that both compound A and compound B have a broad safety range, while compound B is much safer than compound A.

### The novel compounds protect inner ear HCs by maintaining H3K4me2 levels in the gentamycin-induced HC damage model

**Figure 3.**
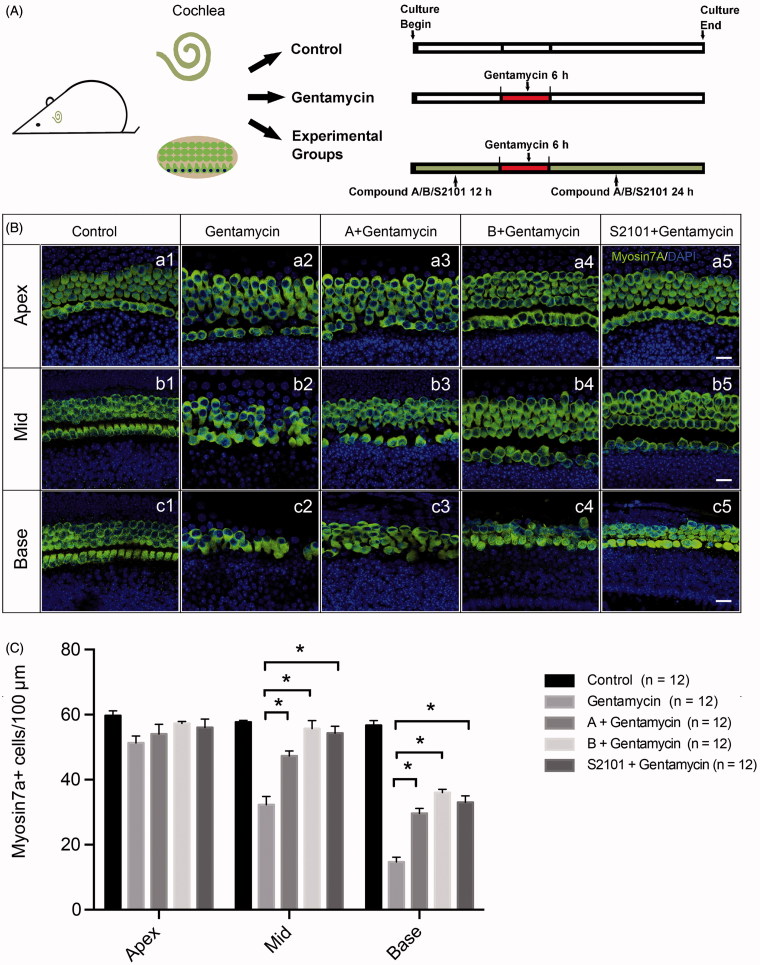
Assessment of HC survival upon gentamycin treatment with or without compound A or B. (A) The schematic diagram of the experiment. (B) Confocal images of the apical, middle, and basal turns in the control (untreated, a1–c1) and gentamycin only (a2–c2) cochlear explant cultures and the compound A (a3–c3), compound B (a4–c4), and S2101 (a5–c5) pretreated cultures. The HCs were labeled with myosin VIIa antibody (green), and the nuclei were stained with DAPI (blue). Scale bar =25 μm. (C) HC counts in the apical, middle, and basal turns of the cochleae from untreated controls, gentamycin-only controls, and pretreatment with compounds A, B, and S2101. Twelve cochleae were used for each group. Data are expressed as the mean ± S.E. * p < .05 vs. the gentamycin-only group by One-Way ANOVA.

We further investigated whether the new compounds can protect mammalian HCs in a gentamycin-induced damage model. The cochlear explants were treated with vehicle alone or with gentamycin only in the untreated and negative control groups, respectively. The experimental groups were pretreated with 20 μM compound A, 20 μM compound B, or 20 μM S2101 for 12 h, then exposed to 1 mM gentamycin for 6 h and allowed to recover for 24 h in the presence of compound A, compound B, or S2101 (Figure 3A). The LSD1 inhibitor S2101 was used as the positive control and has proven to be protective of inner ear HCs and spiral ganglion cells (He et al., 2015; Li et al., 2015a). After treatment, the explants were fixed and stained with myosin VIIa antibody to identify the HCs. The numbers of surviving HCs across the three turns of the organ of Corti were counted. Gentamycin exposure caused a significant reduction in the number of HCs in the middle and basal turns of the gentamycin-only treated cochleae compared to the untreated control group (Figure 3B (b1, b2; c1, c2)). In contrast, pretreatment with 20 μM compound A or compound B significantly reduced gentamycin-induced HC death in the middle and basal turns compared to the gentamycin-only controls (Figure 3B (b2–b4, c2–c4)). These results were similar to the S2101 treatment, which provided significant protection of the HCs against gentamycin-induced damage in the middle and basal turns of the organ of Corti (Figure 3B (b5, c5)). Figure 3(C) shows the quantification of HCs in the middle and basal turns of the cochlear tissues in the different treatment groups (*p* < .05). These results showed that the new compounds have the ability to protect HCs against gentamycin-induced damage. No apparent HC loss was observed in the apical turn in any of the four groups compared to the untreated control group, suggesting that the HCs in the apex of the cochlea are less susceptible to gentamycin-induced damage than those in the middle and basal turns (Figure 3B (a1–a5) and 3(C)). This result is similar to results of previous studies showing that the HCs in the apex of the cochlea are less susceptible to aminoglycoside-induced damage than those in the basal turn (Richardson & Russell, 1991; He et al., 2015; Li et al., 2015a).

**Figure 4.**
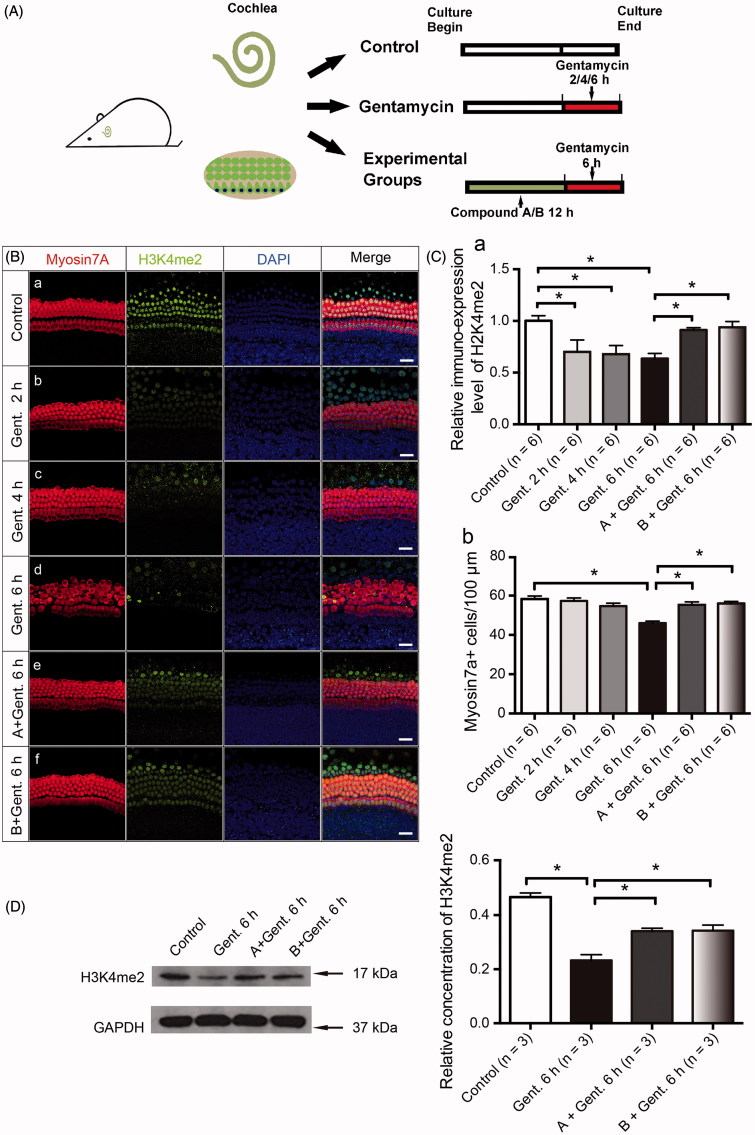
Compounds A and B maintain the level of H3K4me2 in the presence of gentamycin. (A) The schematic diagram of the experiment. (B) The changes in global H3K4 dimethylation (H3K4me2) levels in response to gentamycin exposure and treatment with the different compounds. The normal levels of H3K4me2 are represented by immunofluorescent staining in untreated HCs (a). The H3K4me2 level decreased compared to the control group at 2 h (b), 4 h (c), and 6 h (d) after exposure to 1 mM gentamycin. After 6 h gentamycin exposure, the relative fluorescence intensity of H3K4me2 was significantly stronger in the cultures pretreated with compound A or B (e, f) compared with the gentamycin-only controls (d). Images were taken from the middle turn. The green spots represent the H3K4me2 signal, the anti-myosin VIIa antibody (red) shows the HCs, and the nuclei are stained with DAPI (blue). Scale bar =25 μm. (C) The semi-quantification of the immunofluorescence of H3K4me2 in the different groups (a). The quantification of HCs in the cochlear explants of the different groups (b). Six cochleae were used for each group. Data are expressed as the mean ± S.E. *p < .05, gentamycin-only group vs. the control group by one-way ANOVA. * *p* < .05, compound treatment groups vs. the gentamycin-only 6 h group by one-way ANOVA. (D) Western blot analysis of H3K4me2 levels in the normal group and the gentamycin-exposed cochlear explants with or without pretreatment with compound A or B. GAPDH was used as the loading control. The mean ± S.E. is shown for three experimental replicates. *p < .05 vs. the gentamycin 6 h group by one-way ANOVA.

H3K4me2 is an important histone marker and plays an important role in regulating gene transcription. It is also critical in the survival of cochlear cells during ototoxic injury. We first examined the expression pattern of H3K4me2 in the cochlear sensory epithelium. The staining was strong in the HC nuclei of the untreated cochlear sensory epithelium (Figure 4Ba). We next determined the H3K4me2 level in the cochlear epithelium following 1 mM gentamycin treatment for different time points (Figure 4A). We observed a decrease in H3K4me2 fluorescence in the organ of Corti after 2 h of incubation with 1 mM gentamycin (Figure 4Bb, Ca). After 4 h, the fluorescence was significantly reduced without apparent HC loss (Figure 4Bc, C (a–b)). There was no statistical significance between the number of HCs in the 2 h and 4 h group, which is in accordance with previous findings in the aminoglycoside damage model (Yu et al., 2013). The western blot results confirmed the decrease in H3K4me2 levels in the organ of Corti following gentamycin damage (Figure 4D, *p* < .05).

The otoprotection of LSD1 inhibitors is mediated through the maintenance of H3K4me2 levels (He et al., 2015; Li et al., 2015a), and we hypothesized that our new compounds with similar chemical structures to LSD1 inhibitors also maintain the level of H3K4me2. We examined the H3K4me2 expression level following compound A and B pretreatment using immunofluorescence staining. The experimental groups were pretreated with 20 μM compound A or 20 μM compound B for 12 h and then exposed to 1 mM gentamycin for 6 h in the presence of compound A or B. The pretreatment with compounds A and B significantly prevented the decrease in H3K4me2 levels compared to gentamycin alone (Figure 4B (d–f), C), and stronger fluorescence signals were seen for both compound A and B compared to the gentamycin-only controls (Figure 4B (e–f), C). These differences were verified by Western blot analysis, indicating that the otoprotection of these new compounds is likely mediated by maintaining H3K4me2 levels in the gentamycin-induced HC damage model (Figure 4D, *p* < .05).

### Treatment with the new compounds prevents gentamycin-induced apoptosis

**Figure 5.**
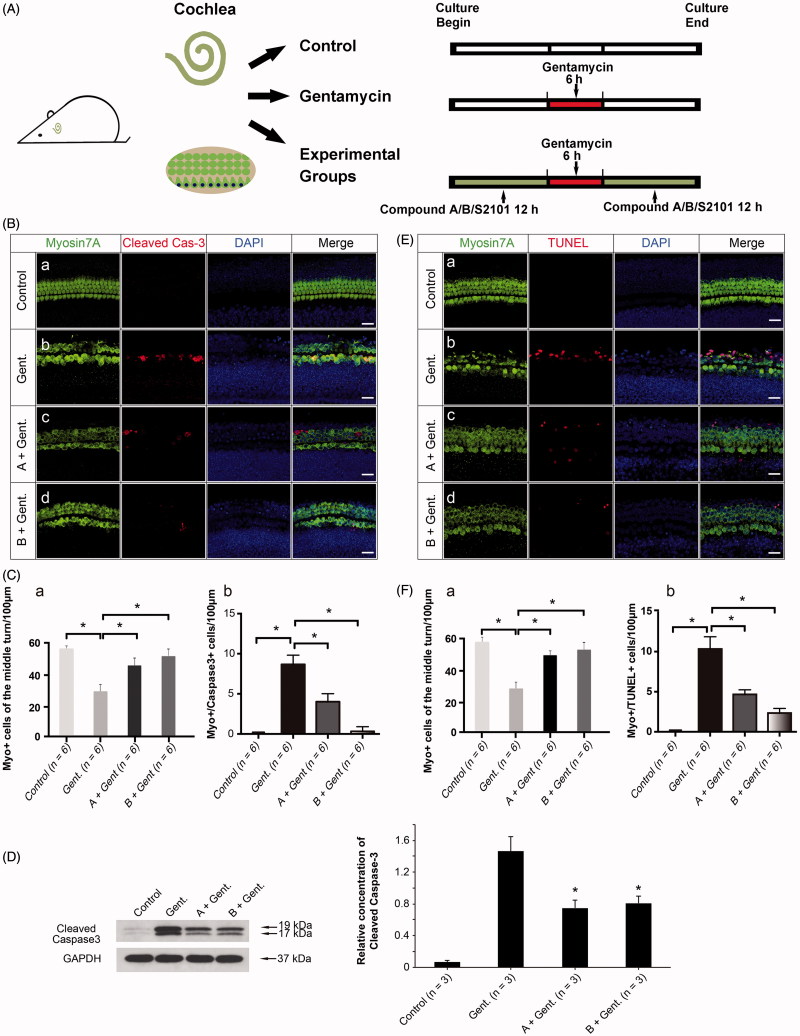
The new compounds reduce apoptosis in the organ of Corti after gentamycin exposure. (A) The schematic diagram of the experiment. (B) The level of cleaved caspase-3 in the organ of Corti decreased in the presence of compound A (c) and compound B (d) when compared with the gentamycin-only group (b). The normal group with no treatment was used as controls (a). (C) The quantification of HCs (a) and myosin VIIa/cleaved caspase-3 double-positive cells (b) in the different groups. Confocal images were taken from the middle turn of each group. The red spots represent the cleaved caspase-3 signal, and anti-myosin VIIa antibody (green) is used as the HC marker. Scale bar =25 μm. Six cochleae were used for each group. Data are expressed as the mean ± S.E. *p < .05 vs. the gentamycin-only group by one-way ANOVA. (D) The relative level of cleaved caspase-3 was measured by Western blot analysis. GAPDH was used as the loading control. All data are shown as the mean ± S.E. for three experimental replicates. *p < .05 vs. the gentamycin-only group by one-way ANOVA. (E) The TUNEL-labeled cells in the organ of Corti decreased in the presence of compound A (c) and compound B (d) when compared with the gentamycin-only controls (b). The normal group with no treatment was used as the control (a). The red spots represent the TUNEL signal. Confocal images were taken from the middle turn of each group. Scale bar =25 μm. (F) The quantification of HCs (a) and myosin VIIa,/TUNEL double-positive cells (b) in the different groups. Six cochleae were used for each group. Data are expressed as the mean ± S.E. *p < .05 vs. the gentamycin-only group by one-way ANOVA.

To assess whether the protection against gentamycin injury by compounds A and B is achieved through the inhibition of apoptosis, we determined the expression of cleaved caspase-3 in the cochlear sensory epithelium after treatment with gentamycin alone or with gentamycin after pretreatment with compound A or B. Cleaved caspase-3 is a late effector of apoptosis and a major mediator of aminoglycoside-induced apoptosis in auditory HCs (Chang et al., 2011). The experimental groups were pretreated with 20 μM compound A or B for 12 h, then exposed to 1 mM gentamycin for 6 h and allowed to recover for 12 h in the presence of compound A or B (Figure 5A). As shown in Figure 5, an increased number of myosin VIIa/cleaved caspase-3 double-positive cells was seen in the gentamycin-only group compared with intact explants (Figure 5B (a–b)), but the double-positive cell number was decreased in the tissues pretreated with compounds A and B (Figure 5B (c–d)). The quantification of HCs and myosin VIIa/cleaved caspase-3 double-positive cells also demonstrated the protective effect of the new compounds through the inhibition of apoptosis (Figure 5C (a–b), *p* < .05). Quantification by western blot analysis confirmed that compound A and B pretreatment decreased cleaved caspase-3 protein expression levels (Figure 5D, *p* < .05). Moreover, we examined the effect of compounds A and B on apoptosis with the terminal deoxynucleotidyl transferase-mediated dUTP nick end labeling (TUNEL) assay. The number of myosin VIIa/TUNEL double-positive cells in the organ of Corti decreased in the presence of compounds A and B when compared with the gentamycin-only control (Figure 5E (b–d)). The quantification of HCs and myosin VIIa/TUNEL double-positive cells confirmed the anti-apoptotic effect of the new compounds (Figure 5F (a–b), *p* < .05). Together with the cleaved-caspase3 results, this indicated that pretreatment with compound A or B might suppress the apoptotic cascade induced by gentamycin.

### The new compounds do not interfere with the uptake of gentamycin or HC function

**Figure 6.**
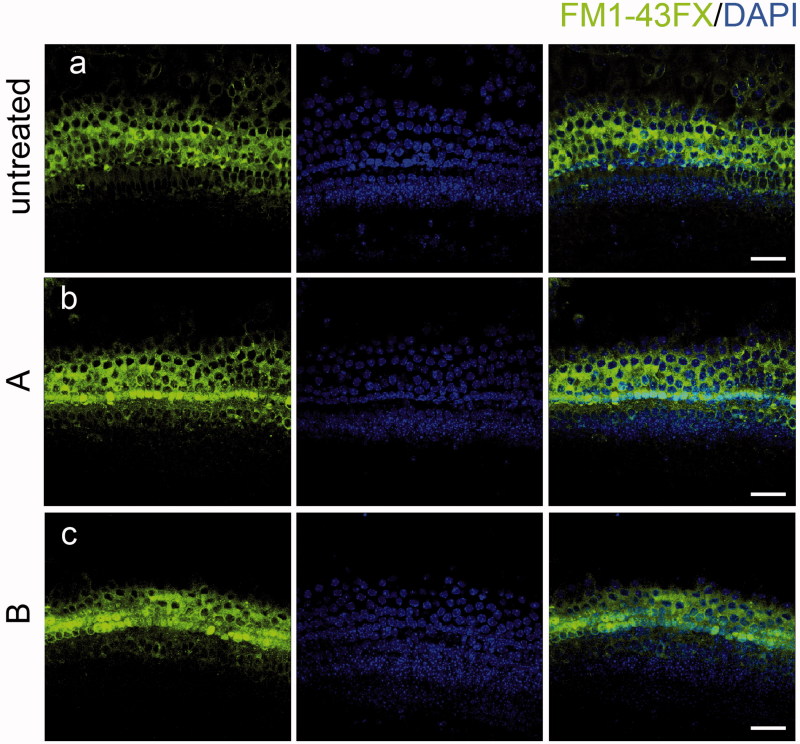
The new compounds do not interfere with FM1-43FX uptake. Compound A and B did not change the uptake of FM1-43FX in the organ of Corti (b, c) compared with the untreated group (a) indicating that HC functions were not affected by exposure to the new compounds. Confocal images were taken from the middle turn of each group. The green fluorescence represents the FM1-43FX signal. Scale bar =20 μm.

Mechanotransduction plays a key role in mediating aminoglycoside entry into HCs. Thus, one possible reason for the resistance to aminoglycosides could be that HCs might have lost their mechanotransduction capability after treatment with compound A or B. We investigated whether these new compounds interfere with aminoglycoside uptake by monitoring the uptake of the membrane-permeable probe FM1-43FX, which is an amphipathic styryl dye that has been reported to enter the sensory HCs via the mechanotransduction channel and is a commonly used tool for studying the function of HCs (Gale et al., 2001). FM1-43FX was efficiently taken up by HCs of the cultured organs of Corti in both the untreated control group (Figure 6a) and in those treated with compound A or B (Figure 6b,c). These data indicated that the new compounds do not prevent gentamycin uptake into HCs.

## Discussion

Epigenetic mechanisms regulate the structure and activity of the genome in response to intracellular and environmental cues that direct cell type-specific gene networks and control cell development and survival (Weaver et al., 2004; Suva et al., 2013; Layman & Zuo, 2014; Roidl & Hacker, 2014). Posttranslational modifications of histones, including methylation, acetylation, phosphorylation, and ubiquitylation, represent important aspects of epigenetic regulation (Strahl & Allis, 2000), and all of these modifications affect chromatin structure and other genome functions such as DNA replication and repair (Berger, 2007; Taverna et al., 2007).

Histone methylation, which occurs on both lysine and arginine residues, is one of the most abundant and dynamic histone modifications. The patterns of H3K4 methylation have been implicated in many nuclear processes such as transcription activation and repression, DNA replication, DNA recombination, and DNA repair (Santos-Rosa et al., 2002; Wood et al., 2005; Bedford & Clarke, 2009; Gupta et al., 2010; Mosammaparast & Shi, 2010). A previous study showed that incomplete methylation of H3K4 generates a set of lineage-specific genes that are transcriptionally poised in pluripotent stem cells and that can become activated during the process of terminal differentiation (Orford et al., 2008). Moreover, the dimethylation of H3K4 is important both for a proper response to DNA-damaging agents and for meiotic differentiation in mammalian and budding yeast systems (Kim & Buratowski, 2009; Faucher & Wellinger, 2010).

Previous studies showed that HDAC activity plays an important role in the regeneration of HCs in the lateral line of zebrafish (He et al., 2014a), and extensive studies have shown that Lgr5 + supporting cells serve as promising progenitors to regenerate HCs (Chai et al., 2011, 2012; Wang et al., 2015; Li et al., 2015b; Li et al., 2016; Ni et al., 2016a, 2016b; Waqas et al., 2016; Wu et al., 2016; Cheng et al., 2017; Lu et al., 2017; Waqas et al., 2017). Recently, researchers have shown that Lgr5 + progenitors can form organoids and can differentiate into HCs in high yields after treatment with a combination of growth factors, glycogen synthase kinase 3β (GSK3β) inhibitor, and the HDAC inhibitor valproic acid (VPA or V) (McLean et al., 2017). Our recent study showed that the lysine methyltransferases G9a and GLP (G9a/GLP) specific inhibitor BIX01294 significantly down-regulates H3K9me2 and decreases HC regeneration after neomycin-induced HC loss through inactivation of the Wnt/β-catenin and Fgf signaling pathways (Tang et al., 2016). All of these results suggest the potential of inner ear HC regeneration through epigenetic regulation.

In a previous study, we found that stable levels of H3K4me2 modification might contribute to the survival of cochlear HCs and SGNs (He et al., 2015; Li et al., 2015a). In the present study, we have developed a novel group of chemical compounds with better water solubility than S2101 that specifically prevent the decrease in H3K4me2 levels that is normally seen after gentamycin exposure. We first examined the ototoxicity of these compounds by incubating them with cultured cochlear explants at different concentrations for 24 h. We found that low concentrations of 20 μM and 40 μM were safe enough in the culture media. At high concentrations of 200 μM, compound A induced significant HC loss while compound B did not. We then measured the H3K4me2 level upon gentamycin-induced HC damage in cultured cochlear explants. We found that gentamycin treatment induced a rapid decrease in the H3K4me2 level in the organ of Corti, which was followed by activation of the apoptotic pathway, and H3K4me2 disappeared after prolonged gentamycin treatment, largely due to the massive loss of HCs. These data are in accordance with the results we have obtained previously for HCs exposed to gentamycin. The use of the two of the new compounds led to increased levels of H3K4me2 and increased HC survival upon exposure to gentamycin compared to cells treated only with gentamycin. A previous report showed that H3K4 methylation is involved in DNA damage responses and has antiapoptotic effects through regulation of transcription factor E2 promoter-binding factor 1 (E2F1) (Tyagi & Herr, 2009), and a recent study suggested that loss of H3K4 dimethylation is sufficient to drive yeast cells into apoptosis, which indicates that H3K4 methylation is an anti-apoptotic marker (Walter et al., 2014). Therefore, it is possible that maintaining the level of H3K4me2 by using the new compounds might lead to the activation or suppression of specific genes, thus preventing the processes that lead to cell death in response to aminoglycoside exposure.

It is believed that apoptotic cell death, instead of necrosis, is the primary cause of HC death induced by aminoglycosides (Selimoglu, 2007; Mangiardi et al., 2004). Apoptosis has also been suggested to be the primary mechanism of HC death in cochlear explant cultures in response to the direct application of aminoglycosides (Cunningham et al., 2002; Taylor et al., 2008). Labeling the activated form of caspase-3 and measuring TUNEL-positive nuclei showed that most HCs die via the classical apoptotic pathway, and we have shown in this study that the caspase-dependent pathway was suppressed by pretreatment with the two new compounds. Our results show that the decrease in H3K4me2 levels in HCs in response to gentamycin exposure was rescued by pretreatment with the new compounds and that this prevented the activation of the apoptotic pathway, but the detailed molecular mechanism through which the new compounds exert their otoprotective effects is still unknown.

Auditory HCs have repeatedly been shown to be susceptible to ototoxicity from a multitude of drugs, and it is known that DNA methylation can repress genes encoding drug metabolizing enzymes, drug transporters, and even drug target genes, which might alter the pharmacokinetics and pharmacodynamics of these ototoxic drugs. The results of our experiments with our novel compounds suggest that the maintenance of H3K4me2 level by the compounds protects against gentamycin-induced HC death in the organ of Corti. Furthermore, administration of these compounds appears to disrupt the onset of apoptosis, and we propose that preventing apoptosis is the general mechanism underlying the otoprotective effects of these compounds against aminoglycoside-induced mammalian HC loss. Such findings provide novel scientific insights into HC damage and might offer an important window to understanding how epigenetic modifications might be manipulated for the purpose of regenerating HCs to restore hearing and vestibular functions. Further investigations are currently in progress to gain a better understanding of the complex gene regulatory networks behind this otoprotective effect in response to gentamycin exposure.
